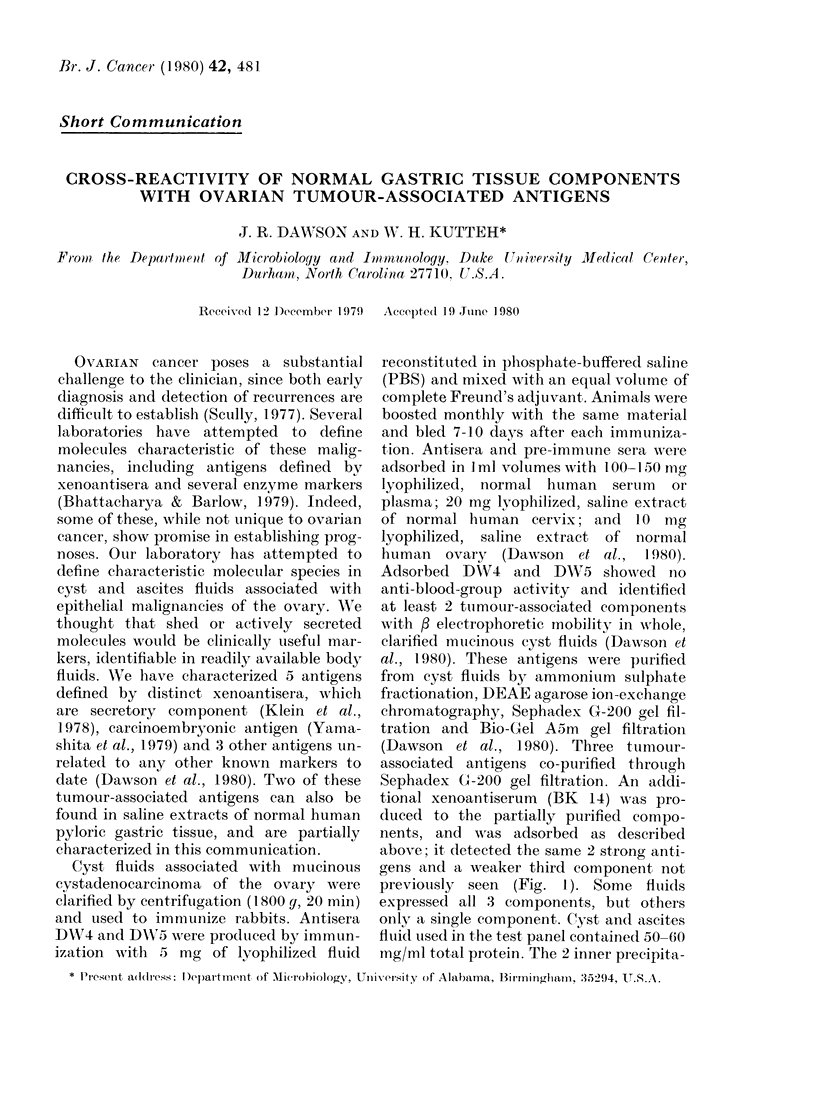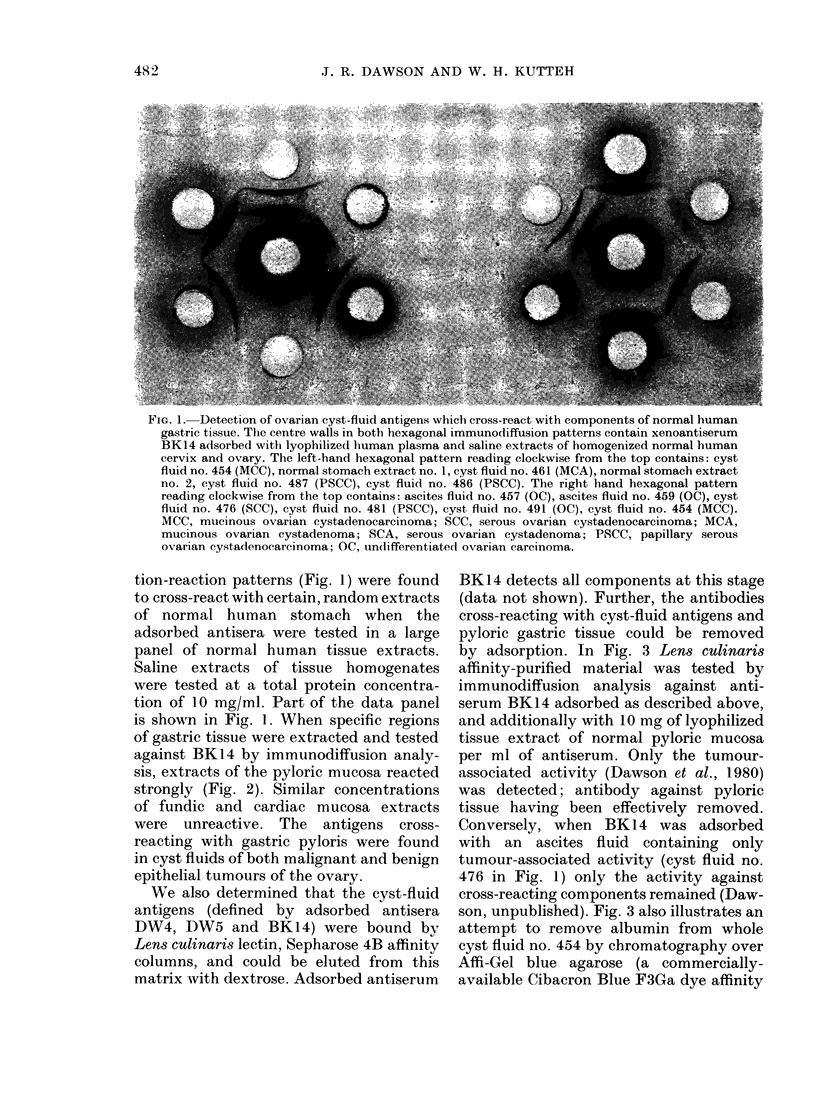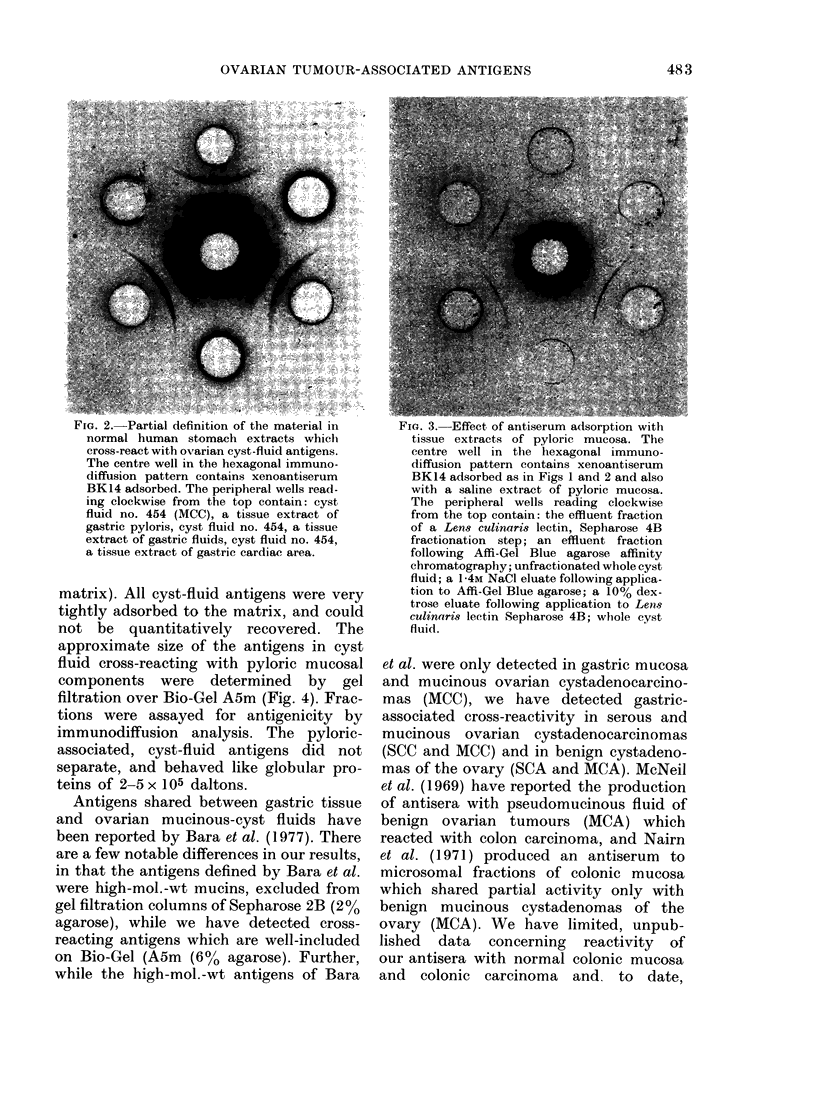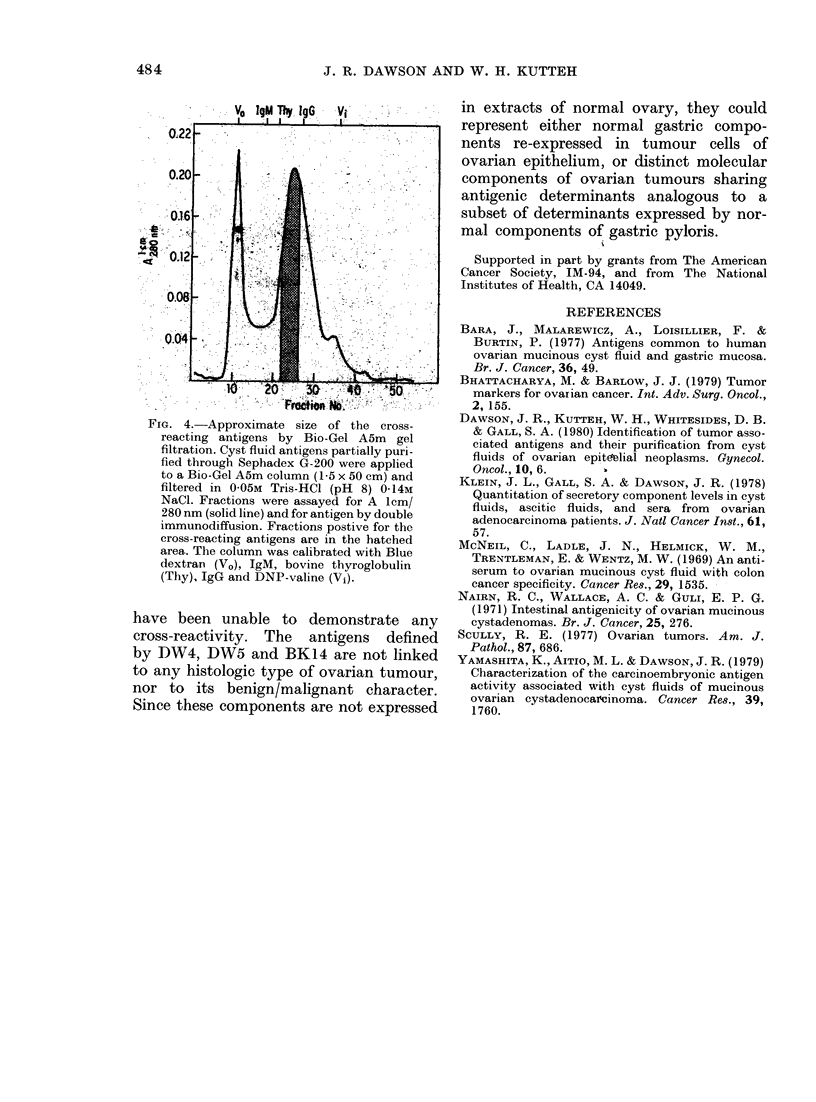# Cross-reactivity of normal gastric tissue components with ovarian tumour-associated antigens.

**DOI:** 10.1038/bjc.1980.262

**Published:** 1980-09

**Authors:** J. R. Dawson, W. H. Kutteh

## Abstract

**Images:**


*Br. J. Cancer* (1980) **42**, 481

***Short Communication***

### CROSS-REACTIVITY OF NORMAL GASTRIC TISSUE COMPONENTS WITH OVARIAN TUMOUR-ASSOCIATED ANTIGENS

J. R. DAWSON AND W. H. KUTTEH*

*From the Department of Microbiology and Immunology, Duke University Medical Center, Durham, North Carolina 27710, U.S.A.*

Received 12 December 1979 Accepted 19 June 1980

OVARIAN cancer poses a substantial challenge to the clinician, since both early diagnosis and detection of recurrences are difficult to establish (Scully, 1977). Several laboratories have attempted to define molecules characteristic of these malignancies, including antigens defined by xenoantisera and several enzyme markers (Bhattacharya & Barlow, 1979). Indeed, some of these, while not unique to ovarian cancer, show promise in establishing prognoses. Our laboratory has attempted to define characteristic molecular species in cyst and ascites fluids associated with epithelial malignancies of the ovary. We thought that shed or actively secreted molecules would be clinically useful markers, identifiable in readily available body fluids. We have characterized 5 antigens defined by distinct xenoantisera, which are secretory component (Klein *et al.*, 1978), carcinoembryonic antigen (Yamashita *et al.*, 1979) and 3 other antigens unrelated to any other known markers to date (Dawson *et al.*, 1980). Two of these tumour-associated antigens can also be found in saline extracts of normal human pyloric gastric tissue, and are partially characterized in this communication.

Cyst fluids associated with mucinous cystadenocarcinoma of the ovary were clarified by centrifugation (1800 *g*, 20 min) and used to immunize rabbits. Antisera DW4 and DW5 were produced by immunization with 5 mg of lyophilized fluid reconstituted in phosphate-buffered saline (PBS) and mixed with an equal volume of complete Freund's adjuvant. Animals were boosted monthly with the same material and bled 7-10 days after each immunization. Antisera and pre-immune sera were adsorbed in 1ml volumes with 100–150 mg lyophilized, normal human serum or plasma; 20 mg lyophilized, saline extract of normal human cervix; and 10 mg lyophilized, saline extract of normal human ovary (Dawson *et al.*, 1980). Adsorbed DW4 and DW5 showed no anti-blood-group activity and identified at least 2 tumour-associated components with $\beta$ electrophoretic mobility in whole, clarified mucinous cyst fluids (Dawson *et al.*, 1980). These antigens were purified from cyst fluids by ammonium sulphate fractionation, DEAE agarose ion-exchange chromatography, Sephadex G-200 gel filtration and Bio-Gel A5m gel filtration (Dawson *et al.*, 1980). Three tumour-associated antigens co-purified through Sephadex G-200 gel filtration. An additional xenoantiserum (BK 14) was produced to the partially purified components, and was adsorbed as described above; it detected the same 2 strong antigens and a weaker third component not previously seen (Fig. 1). Some fluids expressed all 3 components, but others only a single component. Cyst and ascites fluid used in the test panel contained 50–60 mg/ml total protein. The 2 inner precipita-

* Present address: Department of Microbiology, University of Alabama, Birmingham, 35294, U.S.A.

FIG. 1.—Detection of ovarian cyst-fluid antigens which cross-react with components of normal human gastric tissue. The centre walls in both hexagonal immunodiffusion patterns contain xenoantiserum BK14 adsorbed with lyophilized human plasma and saline extracts of homogenized normal human cervix and ovary. The left-hand hexagonal pattern reading clockwise from the top contains: cyst fluid no. 454 (MCC), normal stomach extract no. 1, cyst fluid no. 461 (MCA), normal stomach extract no. 2, cyst fluid no. 487 (PSCC), cyst fluid no. 486 (PSCC). The right hand hexagonal pattern reading clockwise from the top contains: ascites fluid no. 457 (OC), ascites fluid no. 459 (OC), cyst fluid no. 476 (SCC), cyst fluid no. 481 (PSCC), cyst fluid no. 491 (OC), cyst fluid no. 454 (MCC). MCC, mucinous ovarian cystadenocarcinoma; SCC, serous ovarian cystadenocarcinoma; MCA, mucinous ovarian cystadenoma; SCA, serous ovarian cystadenoma; PSCC, papillary serous ovarian cystadenocarcinoma; OC, undifferentiated ovarian carcinoma.

tion-reaction patterns (Fig. 1) were found to cross-react with certain, random extracts of normal human stomach when the adsorbed antisera were tested in a large panel of normal human tissue extracts. Saline extracts of tissue homogenates were tested at a total protein concentration of 10 mg/ml. Part of the data panel is shown in Fig. 1. When specific regions of gastric tissue were extracted and tested against BK14 by immunodiffusion analysis, extracts of the pyloric mucosa reacted strongly (Fig. 2). Similar concentrations of fundic and cardiac mucosa extracts were unreactive. The antigens cross-reacting with gastric pyloris were found in cyst fluids of both malignant and benign epithelial tumours of the ovary.

We also determined that the cyst-fluid antigens (defined by adsorbed antisera DW4, DW5 and BK14) were bound by *Lens culinaris* lectin, Sepharose 4B affinity columns, and could be eluted from this matrix with dextrose. Adsorbed antiserum BK14 detects all components at this stage (data not shown). Further, the antibodies cross-reacting with cyst-fluid antigens and pyloric gastric tissue could be removed by adsorption. In Fig. 3 *Lens culinaris* affinity-purified material was tested by immunodiffusion analysis against antiserum BK14 adsorbed as described above, and additionally with 10 mg of lyophilized tissue extract of normal pyloric mucosa per ml of antiserum. Only the tumour-associated activity (Dawson *et al.*, 1980) was detected; antibody against pyloric tissue having been effectively removed. Conversely, when BK14 was adsorbed with an ascites fluid containing only tumour-associated activity (cyst fluid no. 476 in Fig. 1) only the activity against cross-reacting components remained (Dawson, unpublished). Fig. 3 also illustrates an attempt to remove albumin from whole cyst fluid no. 454 by chromatography over Affi-Gel blue agarose (a commercially-available Cibacron Blue F3Ga dye affinity

FIG. 2.—Partial definition of the material in normal human stomach extracts which cross-react with ovarian cyst-fluid antigens. The centre well in the hexagonal immunodiffusion pattern contains xenoantiserum BK14 adsorbed. The peripheral wells reading clockwise from the top contain: cyst fluid no. 454 (MCC), a tissue extract of gastric pyloris, cyst fluid no. 454, a tissue extract of gastric fluids, cyst fluid no. 454, a tissue extract of gastric cardiac area.

FIG. 3.—Effect of antiserum adsorption with tissue extracts of pyloric mucosa. The centre well in the hexagonal immunodiffusion pattern contains xenoantiserum BK14 adsorbed as in Figs 1 and 2 and also with a saline extract of pyloric mucosa. The peripheral wells reading clockwise from the top contain: the effluent fraction of a *Lens culinaris* lectin, Sepharose 4B fractionation step; an effluent fraction following Affi-Gel Blue agarose affinity chromatography; unfractionated whole cyst fluid; a 1·4M NaCl eluate following application to Affi-Gel Blue agarose; a 10% dextrose eluate following application to *Lens culinaris* lectin Sepharose 4B; whole cyst fluid.

matrix). All cyst-fluid antigens were very tightly adsorbed to the matrix, and could not be quantitatively recovered. The approximate size of the antigens in cyst fluid cross-reacting with pyloric mucosal components were determined by gel filtration over Bio-Gel A5m (Fig. 4). Fractions were assayed for antigenicity by immunodiffusion analysis. The pyloric-associated, cyst-fluid antigens did not separate, and behaved like globular proteins of $2\text{–}5 \times 10^5$ daltons.

Antigens shared between gastric tissue and ovarian mucinous-cyst fluids have been reported by Bara *et al.* (1977). There are a few notable differences in our results, in that the antigens defined by Bara *et al.* were high-mol.-wt mucins, excluded from gel filtration columns of Sepharose 2B (2% agarose), while we have detected cross-reacting antigens which are well-included on Bio-Gel (A5m (6% agarose). Further, while the high-mol.-wt antigens of Bara *et al.* were only detected in gastric mucosa and mucinous ovarian cystadenocarcinomas (MCC), we have detected gastric-associated cross-reactivity in serous and mucinous ovarian cystadenocarcinomas (SCC and MCC) and in benign cystadenomas of the ovary (SCA and MCA). McNeil *et al.* (1969) have reported the production of antisera with pseudomucinous fluid of benign ovarian tumours (MCA) which reacted with colon carcinoma, and Nairn *et al.* (1971) produced an antiserum to microsomal fractions of colonic mucosa which shared partial activity only with benign mucinous cystadenomas of the ovary (MCA). We have limited, unpublished data concerning reactivity of our antisera with normal colonic mucosa and colonic carcinoma and, to date,

FIG. 4.—Approximate size of the cross-reacting antigens by Bio-Gel A5m gel filtration. Cyst fluid antigens partially purified through Sephadex G-200 were applied to a Bio-Gel A5m column (1·5 × 50 cm) and filtered in 0·05M Tris-HCl (pH 8) 0·14M NaCl. Fractions were assayed for A 1cm/280 nm (solid line) and for antigen by double immunodiffusion. Fractions postive for the cross-reacting antigens are in the hatched area. The column was calibrated with Blue dextran ($V_0$), IgM, bovine thyroglobulin (Thy), IgG and DNP-valine ($V_i$).

have been unable to demonstrate any cross-reactivity. The antigens defined by DW4, DW5 and BK14 are not linked to any histologic type of ovarian tumour, nor to its benign/malignant character. Since these components are not expressed in extracts of normal ovary, they could represent either normal gastric components re-expressed in tumour cells of ovarian epithelium, or distinct molecular components of ovarian tumours sharing antigenic determinants analogous to a subset of determinants expressed by normal components of gastric pyloris.

Supported in part by grants from The American Cancer Society, IM-94, and from The National Institutes of Health, CA 14049.

### REFERENCES

BARA, J., MALAREWICZ, A., LOISILLIER, F. & BURTIN, P. (1977) Antigens common to human ovarian mucinous cyst fluid and gastric mucosa. *Br. J. Cancer*, **36**, 49.

BHATTACHARYA, M. & BARLOW, J. J. (1979) Tumor markers for ovarian cancer. *Int. Adv. Surg. Oncol.*, **2**, 155.

DAWSON, J. R., KUTTEH, W. H., WHITESIDES, D. B. & GALL, S. A. (1980) Identification of tumor associated antigens and their purification from cyst fluids of ovarian epitelial neoplasms. *Gynecol. Oncol.*, **10**, 6.

KLEIN, J. L., GALL, S. A. & DAWSON, J. R. (1978) Quantitation of secretory component levels in cyst fluids, ascitic fluids, and sera from ovarian adenocarcinoma patients. *J. Natl Cancer Inst.*, **61**, 57.

MCNEIL, C., LADLE, J. N., HELMICK, W. M., TRENTLEMAN, E. & WENTZ, M. W. (1969) An antiserum to ovarian mucinous cyst fluid with colon cancer specificity. *Cancer Res.*, **29**, 1535.

NAIRN, R. C., WALLACE, A. C. & GULI, E. P. G. (1971) Intestinal antigenicity of ovarian mucinous cystadenomas. *Br. J. Cancer*, **25**, 276.

SCULLY, R. E. (1977) Ovarian tumors. *Am. J. Pathol.*, **87**, 686.

YAMASHITA, K., AITIO, M. L. & DAWSON, J. R. (1979) Characterization of the carcinoembryonic antigen activity associated with cyst fluids of mucinous ovarian cystadenocarcinoma. *Cancer Res.*, **39**, 1760.